# The post-hospitalization huddle: An interprofessional education model for clinical telemedicine

**DOI:** 10.1016/j.xjep.2025.100774

**Published:** 2025-11-06

**Authors:** Jessica Thayer, Brett Miller, Marcelino Mederos Liriano, Kathryn L. Hoffman, Jesse Thompson, Gina Baugh, Jenna Sizemore

**Affiliations:** a School of Medicine, West Virginia University, USA; b Academic Personnel, Health Sciences Center, West Virginia University, USA; c School of Pharmacy, West Virginia University, Health Sciences Center, Introductory Pharmacy Practice Experiences, Office of Experiential Learning, USA

## Abstract

To address the growing need for telemedicine experience and interprofessional collaboration among early learners, an Interprofessional Education (IPE) Huddle was developed and integrated into a telemedicine-based outpatient clinic. This clinic serves patients at risk for geographic healthcare disparities post-hospital discharge. The IPE Huddles provided pre-encounter discussions on-site at the clinic just prior to a patient encounter. The Huddles facilitated team-based planning and allowed students to contribute to patient care during virtual visits. Participants from diverse health professions were assessed via the Interprofessional Collaborative Competencies Attainment Survey (ICCAS) and a telemedicine exposure survey. Results demonstrated significant improvement in interprofessional competencies and highlighted the value of interprofessional collaboration in virtual clinical settings.

**Format:**

Our experience was delivered in a clinical setting with a small group of learners completing structured huddle discussions.

## 1. Target audience

Prelicensure or early clinical health professional students from a variety of disciplines, including medicine, pharmacy, physical therapy, physician assistant, and public health (n = 21) were deployed in interprofessional groups to assist with the coordinated care of patients enrolled in the transitions of care clinic. Most students were in the clinical stage of their professional training.

## 2. Objectives

1. Deploy interprofessional student teams from multiple disciplines in interprofessional huddles.
2. Collaborate with different health professions to develop a team-based approach for patients at risk of geographic health disparities.
3. Integrate team-based approaches for clinical encounters for health sciences students for patients with low ambulatory access.

## 3. Activity description

The Interprofessional Education (IPE) Huddle and Intensive Telemedicine Transitions of Care Clinic (I-TTC) were developed as part of a three-fold approach to interprofessional practice and education at our institution. Early learners took part in a yearlong foundational IPE curriculum focused on the four core competencies of IPE, including values and ethics, roles and responsibilities, interprofessional communication, and teamwork.^1^ Next, learners participated in a novel simulation, InterDisciplinary Education Apartment Simulation (IDEAS) which focused on the transition of care from the inpatient to the outpatient setting.^2^ Students then had the opportunity to voluntarily practice team-based skills in our Transitions of Care Clinic through the IPE Huddle and I-TTC.

In our Appalachian patient population, several patients face significant barriers to post-hospitalization outpatient healthcare access. The I-TTC was created to address this gap in care for patients, while also emphasizing the need for team-based care with the growing use of telemedicine. The IPE Huddle was embedded into the clinic to incorporate telemedicine education of health professions students, while working towards the common goal of patient care after hospital discharge. This method of collaboration was designed to provide multiple angles of insight into several facets of patient care, including medication regimen optimization, navigating barriers to healthcare access, mobility and home safety assessments, as well as patient education.

Due to the varied educational backgrounds of our learners and their voluntary participation, they were not required to have prior telemedicine experiences or complete pre-requisite reading prior to the patient encounters. Learners were encouraged to have participated in the IDEAS project but not required. Participants were recruited from the academic center’s schools of health professions, including the School of Medicine (SOM), which encompasses programs in medicine, physical therapy, occupational therapy, and physician assistant studies, as well as the School of Nursing (SON), School of Pharmacy (SOP), and School of Public Health (PH). The telemedicine clinic was established through an internal departmental award designed to expand access to care for patients with limited ambulatory services following a hospitalization. This initiative created a structured model of multiple virtual follow-up visits starting within two weeks of hospital discharge and incorporated the use of home monitoring medical devices to support the management of chronic health conditions.^3^ A total of thirty-one visits were completed and students participated in approximately a third of these encounters allowing them to gain experience in telemedicine assessment, chronic disease management, and interdisciplinary care. Additional encounters without a huddle were conducted by the supervising physician as needed based on patient schedule and medical needs.

Learners completed an “IPE Huddle” form for each participating patient at the beginning of each scheduled meeting, which included a short summary of the patient’s hospitalization, the most recent medication list, and known follow-up appointments (Supplement A). The form was left blank for the student team to complete, and approximately thirty minutes were set aside for the huddle. Students were not expected to complete any preparatory work prior to the huddle. A group of three attending physicians volunteered to facilitate all huddles and subsequent patient encounters. Huddles occurred if at least one attending was able to be present but often, all three were there to assist learner teams. Learners participated in at least one patient encounter but often one to three patients were discussed per “IPE Huddle” experience. Learners participated with patient consent in the telemedicine visit as appropriate for their profession and completed direct patient care tasks such as obtaining patient history, performing medication reconciliations, and patient education based on the completed huddle.

## 4. Assessment

Learners evaluated the IPE Huddle experience using the 20-item Interprofessional Collaborative Competencies Attainment Survey (ICCAS) (Supplement B). The ICCAS is a validated instrument that measures self-reported changes in interprofessional collaborative competencies across domains such as communication, collaboration, roles and responsibilities, patient/family-centered care, conflict management, and team functioning.^4^ Participants also completed a retrospective pre/post Telemedicine Survey (Supplement C) administered on a voluntary basis after their telemedicine patient encounters. This survey assessed their knowledge of telemedicine concepts and tools, their confidence in performing clinical tasks through telemedicine, and their outlook on the role of telemedicine in reducing healthcare disparities and supporting future practice. The Telemedicine Survey is a non-validated survey designed to assess institutional exposure to telemedicine across different health professions to guide further curricular development. Formative feedback was given to each huddle by the supervising physician after the patient encounter, using insights from the patient experience, as well as any relevant information from a follow-up visit. Data was collected using LearningSpace software (Elevate Healthcare). Statistical analysis was performed on ICCAS survey results using Wilcox signed rank test with a p value of <0.05, used to determine statistical significance. Given the low yield of completed telemedicine surveys, statistical analysis was not performed on the results.

## 5. Evaluation

All but two learners participated in the IDEAS Simulation (n = 19), with over half completing it prior to their “IPE Huddle” (n = 12). In total, twenty-one volunteer learners participated from the MD, PA, PT, PH, and SOP programs, the majority of whom were pharmacy students (n = 16). Every participant completed the ICCAS with two participants leaving some questions unanswered. This resulted in eight questions having a reduced total number to 20 responses. All ICCAS survey questions and subclasses were found to be significantly different (p < 0.05 and p < 0.001 respectively) following participation in IPE telemedicine huddles (Table 1). Regarding the Telemedicine Survey, six learners completed all questions retrospectively regarding their perceptions before and after the IPE telemedicine huddle. Given the low yield, there was no statistical difference noted in answers following participation, although the average score differences following participation compared to before were notable in questions evaluating learners general understanding of telemedicine (4.67 [0.52 sd], 3.67 [0.82 sd]), the effectiveness of telemedicine performed in a multidisciplinary team setting (4.33 [0.52 sd], 3.67 [0.52 sd]), and plans to incorporate telemedicine into their future practice(4.50 [0.84 sd], 3.83 [0.41 sd]).

## 6. Impact

Through the implementation of our “IPE Huddles,” learners from health professions conducted telemedicine clinical encounters and contributed to team-based interventions for patients at risk for barriers to healthcare access during an ongoing pandemic. Most learners reported this was their first experience with clinical telemedicine encounters providing a necessary educational experience, especially with heightened need for clinical telemedicine education. We found statistical differences in all subclasses and individual ICCAS questions following our IPE experience. The Telemedicine Survey was underpowered in large part due to inconsistent awareness of learners needing to complete both surveys and time constraints following the educational experience which prevented learners from completing surveys in the clinical space. However, results of this survey pointed to learners gaining an understanding of the field of telemedicine as well as its effectiveness when performed in multidisciplinary team setting with plans to use it in future practice. Additionally, the results of the Telemedicine Survey were given to leadership in participating schools to guide future telemedicine curricula. In large part, we feel the structure of the IPE Huddle prior to the clinical encounter provided a dialogue for learners to not only discuss the case clinically but created a foundation for team-based participation.

Although during this activity students still met at the clinic due to institutional requirements of telemedicine, the broader integration of telemedicine into interprofessional education has the potential to ease common barriers such as scheduling conflicts and transportation challenges for both learners and patients.^5^ It would likely be feasible to perform these huddles virtually with learners and faculty prior to clinical encounters. With the growing use of telemedicine in clinical practice, there is a strong need to incorporate telemedicine training into all health professions curricula. Several institutions have begun establishing and refining telemedicine curricula in response to these growing demands, with success often measured by positive participant survey responses.^6,7^ Given that hospital discharge places patients at risk for medical vulnerability, especially those patients with barriers to healthcare access, we feel more clinical IPE can be effective in the post-hospitalization setting.^5^

Telemedicine can be an ideal opportunity to increase healthcare access for rural patients, as many cite commute distance as a major factor in appointment attendance.^6^ This can be an ideal health delivery model as the scarcity of hospital access is significantly more pronounced due to longer commute distances and financial barriers to access healthcare.^8^ The complex care needs of the recently discharged patient can benefit from interprofessional care. The next generations of students will likely expect continued telemedicine participation in an interprofessional setting, and this framework of an IPE huddle can provide structure to those encounters. Interventions crafted by learners are likely to have lasting impacts on health outcomes, revealing the power of using interprofessional education as a tool not only for student learning, but for improved patient care. In addition, the advent of this telemedicine IPE experience may increase patient access to team-based health care which they would have little access to locally.

## Supplementary Material

Appendix A. Example “IPE Huddle” Template

Appendix B. IPE TCC ICCAS (2024)

Appendix C. Telemedicine Survey

Appendix A. Supplementary data

Supplementary data to this article can be found online at https://doi.org/10.1016/j.xjep.2025.100774.

## Figures and Tables

**Table 1:** ICCAS.

Tab 2. ICCAS Subscales	Individual Measures	N	Pre	Post	Mean Diff	95 % CI	P-value

**Communication**	**Mean Score**		**5.76**	**6.33**	**0.58**	**0.43–0.73**	**<0.001**
1	Communicate	21	5.57	6.38	0.81	0.47–1.15	<0.001
2	Listen	21	6.43	6.71	0.29	0.03–0.54	0.030
3	Judge	21	5.95	6.52	0.57	0.20–0.94	0.004
4	Feedback	20	5.30	6.00	0.70	0.30–1.10	0.018
5	Express	21	5.52	6.05	0.52	0.15–0.89	0.007
**Collaboration**	**Mean Score**		**5.67**	**6.33**	**0.67**	**0.47–0.86**	**<0.001**
6	Address	21	5.33	6.05	0.71	0.36–1.07	<0.001
7	Enhance	21	5.86	6.48	0.62	0.25–0.99	0.002
8	Learn	21	5.81	6.48	0.67	0.33–1.00	<0.001
**Roles and Responsibilities**	**Mean Score**		**5.43**	**6.28**	**0.85**	**0.63–1.08**	**<0.001**
9	Identify	21	5.38	6.29	0.90	0.50–1.31	<0.001
10	Account	20	5.65	6.25	0.60	0.13–1.07	0.014
11	Understand	20	5.30	6.15	0.85	0.36–1.34	0.002
12	Recognize	21	5.38	6.43	1.05	0.52–1.58	<0.001
**Collaborative patient/family centered approach**	**Mean Score**		**5.42**	**6.23**	**0.82**	**0.50–1.13**	**<0.001**
13	Assess	20	5.20	6.20	1.00	0.41–1.59	0.002
14	Provide	20	5.50	6.30	0.80	0.28–1.32	0.004
15	Include	20	5.55	6.20	0.65	0.04–1.26	0.040
**Conflict management/Resolution**	**Mean Score**		**5.80**	**6.36**	**0.56**	**0.33–0.79**	**<0.001**
16	Respect	20	6.05	6.50	0.45	0.13–0.77	0.009
17	Perspective	21	5.81	6.48	0.67	0.18–1.15	0.009
18	Ideas	20	5.55	6.10	0.55	0.11–0.99	0.017
**Team Functioning**	**Mean Score**		**5.47**	**6.32**	**0.85**	**0.51–1.20**	**<0.001**
19	Plan	21	5.38	6.24	0.86	0.26–1.46	0.007
20	Responsibility	20	5.55	6.40	0.85	0.47–1.23	<0.001

ICCAS: Interprofessional Collaborative Competencies Attainment Survey.
