# Supplementary material for: The post-hospitalization huddle: An interprofessional education model for clinical telemedicine
Source: J Interprof Educ Pract. Author manuscript; Available in PMC 2025 Dec 17. (PMC12707798; doi:10.1016/j.xjep.2025.100774)
Supplement: Appendix A. Example “IPE Huddle” Template [file NIHMS2121538-supplement-Appendix_A__Example__IPE_Huddle__Template.pdf]

Appendix A. **Example "IPE Huddle" Template**

Brief HPI: *39 yo male with no PMH or prior hospitalizations who initially presented with abdominal distension, pain, and non-bloody vomiting. He had a complicated hospitalization which included an ICU stay and severe anemia requiring massive transfusion protocol. On evaluation he was found to have decompensated cirrhosis likely secondary to heavy alcohol use per family. Multiple esophageal varices were found on EGD without any acute bleeding noted at time of procedure. His anemia stabilized and he was transferred out of the ICU on a PPI and steroid therapy for a 28-day course. Shortly after, he was discharged home with new medications for further cirrhosis management, psychiatry follow-up for continued alcohol cessation and hepatology for evaluation of liver transplant candidacy. On initial post-discharge follow-up phone call patient described difficulty obtaining Rifaximin due to insurance coverage and was not taking prednisolone due to it being "similar to another medication he was taking." Otherwise had some minor epistaxis but no other complaints. He stated he was aware of both upcoming GI and PCP follow-up.*

Medications:

- *Campral (Acamprosate) 666mg three times a day*
- *Acetaminophen (Tylenol) as needed*
- *Lasix (Furosemide) 40mg daily*
- *Aldactone (Spironolactone) 100mg daily*
- *Lactulose 15ml daily*
- *Rifaximin 550mg twice a day*
- *Bactrim (Trimethoprim-Sulfamethoxazole) 160mg daily*
- *Prenatal vitamin*
- *Thiamine 100mg daily*
- *Protonix (Pantoprazole) 40mg daily*
- *Prednisolone 40mg daily (completed 7 days prior to discharge) until 4/1 (28 days total)*
- *Prednisolone 30mg daily for 5 days until 4/8*
- *Prednisolone 20mg daily for 5 days until 4/13*
- *Prednisolone 9.9mg daily for 5 days until 4/17 then discontinue*

Barriers: *At risk for low healthcare literacy, no prior PCP or medications, distance from PCP and follow-up, healthcare costs, transportation difficulties*

Current needs: *Refills on Lactulose, Aldactone, Protonix.*

Visit Plan: *Discuss appropriate Tylenol dosing. Discuss steroid taper with dates. Discuss immunizations, BP and weights*

Completed Interventions: *Immunizations recommended with order placed. Will Continue BP checks, answered medication questions, refilled medications, ordered repeat EGD*
