# Supplementary material for: The post-hospitalization huddle: An interprofessional education model for clinical telemedicine
Source: J Interprof Educ Pract. Author manuscript; Available in PMC 2025 Dec 17. (PMC12707798; doi:10.1016/j.xjep.2025.100774)
Supplement: Appendix B. IPE TCC ICCAS (2024) [file NIHMS2121538-supplement-Appendix_B__IPE_TCC_ICCAS__2024_.pdf]

CAE LearningSpace™ - Case Manager

IPE TCC ICCAS (2024)

Case Print

☐ Basic properties
☐ Custom properties
☑ Post-Encounter Learner

Post-Encounter Learner

Interprofessional Collaborative Competency Attainment Survey (POST)

1. Please select your discipline:	( ) Audiology
	( ) Dental Hygiene
	( ) Dentistry
	( ) Medicine
	( ) Music Therapy
	( ) Nursing
	( ) Occupational Therapy
	( ) Pharmacy
	( ) Physical Therapy
	( ) Physician Assistant
	( ) Public Health
	( ) Social Work
	( ) Speech Language Pathology
Please answer the following questions by filling in the circle that most accurately reflects your opinion about the following interprofessional collaboration statements (1 = strongly disagree, 4 = neutral, 7 = strongly agree)	
2. **Before** participating in the learning activities, I was able to promote effective communication among member of an interprofessional (IPE) team*:<br><br>*The patient's family or significant other, when appropriate, are part of the IP team	( ) 1
	( ) 2
	( ) 3
	( ) 4
	( ) 5
	( ) 6
	( ) 7
	( ) N/A
3. **After** participating in the learning activities, I am able to promote effective communication among member of an interprofessional (IPE) team*:<br><br>*The patient's family or significant other, when appropriate, are part of the IP team	( ) 1
	( ) 2
	( ) 3
	( ) 4
	( ) 5
	( ) 6
	( ) 7
	( ) N/A
4. **Before** participating in the learning activities, I was able to actively listen to IP team members' ideas and concerns.	( ) 1
	( ) 2
	( ) 3
	( ) 4
	( ) 5
	( ) 6
	( ) 7
	( ) N/A
5. **After** participating in the learning activities, I am able to actively listen to IP team members' ideas and concerns.	( ) 1
	( ) 2
	( ) 3
	( ) 4
	( ) 5
	( ) 6
	( ) 7
	( ) N/A
6. **Before** participating in the learning activities, I was able to express my ideas and concerns without being judgmental.	( ) 1
	( ) 2
	( ) 3
	( ) 4
	( ) 5
	( ) 6
	( ) 7
	( ) N/A
7. **After** participating in the learning activities, I am able to express my ideas and concerns without being judgmental.	( ) 1
	( ) 2
	( ) 3
	( ) 4
	( ) 5
	( ) 6

	( ) 7
	( ) N/A
8. **Before** participating in the learning activities, I was able to provide constructive feedback to IP team members.	( ) 1
	( ) 2
	( ) 3
	( ) 4
	( ) 5
	( ) 6
	( ) 7
	( ) N/A
9. **After** participating in the learning activities, I am able to provide constructive feedback to IP team members.	( ) 1
	( ) 2
	( ) 3
	( ) 4
	( ) 5
	( ) 6
	( ) 7
	( ) N/A
10. **Before** participating in the learning activities, I was able to express my ideas and concerns in a clear, concise manner.	( ) 1
	( ) 2
	( ) 3
	( ) 4
	( ) 5
	( ) 6
	( ) 7
	( ) N/A
11. **After** participating in the learning activities, I am able to express my ideas and concerns in a clear, concise manner.	( ) 1
	( ) 2
	( ) 3
	( ) 4
	( ) 5
	( ) 6
	( ) 7
	( ) N/A
12. **Before** participating in the learning activities, I was able to seek out IP team members to address issues.	( ) 1
	( ) 2
	( ) 3
	( ) 4
	( ) 5
	( ) 6
	( ) 7
	( ) N/A
13. **After** participating in the learning activities, I am able to seek out IP team members to address issues.	( ) 1
	( ) 2
	( ) 3
	( ) 4
	( ) 5
	( ) 6
	( ) 7
	( ) N/A
14. **Before** participating in the learning activities, I was able to work effectively with IP team members to enhance care.	( ) 1
	( ) 2
	( ) 3
	( ) 4
	( ) 5
	( ) 6
	( ) 7
	( ) N/A
15. **After** participating in the learning activities, I am able to work effectively with IP team members to enhance care.	( ) 1
	( ) 2
	( ) 3
	( ) 4
	( ) 5
	( ) 6
	( ) 7
	( ) N/A
16. **Before** participating in the learning activities, I was able to learn with, from, and about IP team members to enhance care.	( ) 1
	( ) 2
	( ) 3
	( ) 4
	( ) 5
	( ) 6
	( ) 7
	( ) N/A

17. **After** participating in the learning activities, I am able to learn with, from, and about IP team members to enhance care.	( ) 1
	( ) 2
	( ) 3
	( ) 4
	( ) 5
	( ) 6
	( ) 7
	( ) N/A
18. **Before** participating in the learning activities, I was able to identify and describe my abilities and contributions to the IP team.	( ) 1
	( ) 2
	( ) 3
	( ) 4
	( ) 5
	( ) 6
	( ) 7
	( ) N/A
19. **After** participating in the learning activities, I am able to identify and describe my abilities and contributions to the IP team.	( ) 1
	( ) 2
	( ) 3
	( ) 4
	( ) 5
	( ) 6
	( ) 7
	( ) N/A
20. **Before** participating in the learning activities, I was able to be accountable for my contributions to the IP team.	( ) 1
	( ) 2
	( ) 3
	( ) 4
	( ) 5
	( ) 6
	( ) 7
	( ) N/A
21. **After** participating in the learning activities, I am able to be accountable for my contributions to the IP team.	( ) 1
	( ) 2
	( ) 3
	( ) 4
	( ) 5
	( ) 6
	( ) 7
	( ) N/A
22. **Before** participating in the learning activities, I was able to understand the abilities and contributions of IP team members.	( ) 1
	( ) 2
	( ) 3
	( ) 4
	( ) 5
	( ) 6
	( ) 7
	( ) N/A
23. **After** participating in the learning activities, I am able to understand the abilities and contributions of IP team members.	( ) 1
	( ) 2
	( ) 3
	( ) 4
	( ) 5
	( ) 6
	( ) 7
	( ) N/A
24. **Before** participating in the learning activities, I was able to recognize how others' skills and knowledge complement and overlap with my own.	( ) 1
	( ) 2
	( ) 3
	( ) 4
	( ) 5
	( ) 6
	( ) 7
	( ) N/A
25. **After** participating in the learning activities, I am able to recognize how others' skills and knowledge complement and overlap with my own.	( ) 1
	( ) 2
	( ) 3
	( ) 4
	( ) 5
	( ) 6
	( ) 7
	( ) N/A

26. **Before** participating in the learning activities, I was able to use an IP team approach with the patient* to assess the health situation.<br><br>*The word "patient" has been employed to represent client, resident, and service users	( ) 1<br>( ) 2<br>( ) 3<br>( ) 4<br>( ) 5<br>( ) 6<br>( ) 7<br>( ) N/A
27. **After** participating in the learning activities, I am able to use an IP team approach with the patient* to assess the health situation.<br><br>* The word "patient" has been employed to represent client, resident, and service users	( ) 1<br>( ) 2<br>( ) 3<br>( ) 4<br>( ) 5<br>( ) 6<br>( ) 7<br>( ) N/A
28. **Before** participating in the learning activities, I was able to use an IP team approach with the patient to provide whole person care.	( ) 1<br>( ) 2<br>( ) 3<br>( ) 4<br>( ) 5<br>( ) 6<br>( ) 7<br>( ) N/A
29. **After** participating in the learning activities, I am able to use an IP team approach with the patient to provide whole person care.	( ) 1<br>( ) 2<br>( ) 3<br>( ) 4<br>( ) 5<br>( ) 6<br>( ) 7<br>( ) N/A
30. **Before** participating in the learning activities, I was able to include the patient/family in decision-making.	( ) 1<br>( ) 2<br>( ) 3<br>( ) 4<br>( ) 5<br>( ) 6<br>( ) 7<br>( ) N/A
31. **After** participating in the learning activities, I am able to include the patient/family in decision-making.	( ) 1<br>( ) 2<br>( ) 3<br>( ) 4<br>( ) 5<br>( ) 6<br>( ) 7<br>( ) N/A
32. **Before** participating in the learning activities, I was able to actively listen to the perspectives of IP team members.	( ) 1<br>( ) 2<br>( ) 3<br>( ) 4<br>( ) 5<br>( ) 6<br>( ) 7<br>( ) N/A
33. **After** participating in the learning activities, I am able to actively listen to the perspectives of IP team members.	( ) 1<br>( ) 2<br>( ) 3<br>( ) 4<br>( ) 5<br>( ) 6<br>( ) 7<br>( ) N/A
34. **Before** participating in the learning activities, I was able to take into account the ideas of IP team members.	( ) 1<br>( ) 2<br>( ) 3<br>( ) 4<br>( ) 5<br>( ) 6<br>( ) 7<br>( ) N/A
35. **After** participating in the learning activities, I am able to take into account the ideas of IP team members.	( ) 1<br>( ) 2

	( ) 3<br>( ) 4<br>( ) 5<br>( ) 6<br>( ) 7<br>( ) N/A
36. **Before** participating in the learning activities, I was able to address team conflict in a respectful manner.	( ) 1<br>( ) 2<br>( ) 3<br>( ) 4<br>( ) 5<br>( ) 6<br>( ) 7<br>( ) N/A
37. **After** participating in the learning activities, I am able to address team conflict in a respectful manner.	( ) 1<br>( ) 2<br>( ) 3<br>( ) 4<br>( ) 5<br>( ) 6<br>( ) 7<br>( ) N/A
38. **Before** participating in the learning activities, I was able to develop an effective care* plan with IP team members.<br><br>*The term "care" includes: intervention, treatment, therapy, evaluation, ect	( ) 1<br>( ) 2<br>( ) 3<br>( ) 4<br>( ) 5<br>( ) 6<br>( ) 7<br>( ) N/A
39. **After** participating in the learning activities, I am able to develop an effective care* plan with IP team members.<br><br>*The term "care" includes: intervention, treatment, therapy, evaluation, ect	( ) 1<br>( ) 2<br>( ) 3<br>( ) 4<br>( ) 5<br>( ) 6<br>( ) 7<br>( ) N/A
40. **Before** participating in the learning activities, I was able to negotiate responsibilities within overlapping scopes of practice.	( ) 1<br>( ) 2<br>( ) 3<br>( ) 4<br>( ) 5<br>( ) 6<br>( ) 7<br>( ) N/A
41. **After** participating in the learning activities, I am able to negotiate responsibilities within overlapping scopes of practice.	( ) 1<br>( ) 2<br>( ) 3<br>( ) 4<br>( ) 5<br>( ) 6<br>( ) 7<br>( ) N/A
