# Supplementary material for: The post-hospitalization huddle: An interprofessional education model for clinical telemedicine
Source: J Interprof Educ Pract. Author manuscript; Available in PMC 2025 Dec 17. (PMC12707798; doi:10.1016/j.xjep.2025.100774)
Supplement: Appendix C. Telemedicine Survey [file NIHMS2121538-supplement-Appendix_C__Telemedicine_Survey.pdf]

CAE LearningSpace™ - Case Manager

Telemedicine Survey ()

Case Print

- [ ] Basic properties
- [ ] Custom properties
- [x] Post-Encounter Learner

Post-Encounter Learner

Research Cover Letter

Dear Participant,

This letter is a request for you to take part in a research project to assess learner perceptions of telemedicine simulation throughout the health science center. This research is conducted by Dr. Jenna Sizemore and Dr. Gina Baugh through WV STEPS. Your participation in this project is greatly appreciated and will take approximately 5 minutes to fill out the attached survey.

Your involvement in this project will be kept as confidential as legally possible. All data will be reported in aggregate (as a whole). You must be 18 years of age or older to participate. We will not ask any information that should lead back to your identity as a participant. Your participation is completely voluntary. By taking this survey you are consenting to be a participant in this research project. You may skip any question that you do not wish to answer and you may discontinue at any time. Your class standing will not be affected if you decide either not to participate or to withdraw. West Virginia University's Institutional Review Board approval of this project is on file.

I hope that you will participate in this research project, as it could be beneficial in improving learner education in telemedicine. Thank you very much for your time. Should you have any questions about this letter or the research project, please feel free to contact Jenna Sizemore at 304-293-6335 or by e-mail at jsizemo3@hsc.wvu.edu.

Thank you for your time and help with this project.

Sincerely,

Jenna Sizemore, MD

Assistant Professor, West Virginia University, Department Medicine, Section of General Internal Medicine

Director, Rural Health Track, WVU IM Residency Program

Associate Program Director, WVU IM Residency Program

1. Did this virtual simulation discuss telemedicine practices prior to the encounter?	( ) Yes
	( ) No
2. Do you have any previous experience with tele-medicine?	( ) Yes
	( ) No

Telemedicine Knowledge

Please rate the following statements based on before and after you participated in the simulation activity identified in the email.	
3. **Before** participating in the learning activities, I had a general understanding of the field of telemedicine.	( ) 1 - Strongly Disagree
	( ) 2 - Disagree
	( ) 3 - Neutral
	( ) 4 - Agree
	( ) 5 - Strongly Agree
4. **After** participating in the learning activities, I have a general understanding of the field of telemedicine.	( ) 1 - Strongly Disagree
	( ) 2 - Disagree
	( ) 3 - Neutral
	( ) 4 - Agree
	( ) 5 - Strongly Agree
5. **Before** participating in the learning activities, I had a general understanding of how telemedicine is performed in my discipline.	( ) 1 - Strongly Disagree
	( ) 2 - Disagree
	( ) 3 - Neutral
	( ) 4 - Agree
	( ) 5 - Strongly Agree
6. **After** participating in the learning activities, I have a general understanding of how telemedicine is performed in my discipline.	( ) 1 - Strongly Disagree
	( ) 2 - Disagree
	( ) 3 - Neutral
	( ) 4 - Agree
	( ) 5 - Strongly Agree
7. **Before** participating in the learning activities, I was familiar with the types of examination tools used in telemedicine.	( ) 1 - Strongly Disagree
	( ) 2 - Disagree

	( ) 3 - Neutral
	( ) 4 - Agree
	( ) 5 - Strongly Agree
8. **After** participating in the learning activities, I am familiar with the types of examination tools used in telemedicine.	( ) 1 - Strongly Disagree
	( ) 2 - Disagree
	( ) 3 - Neutral
	( ) 4 - Agree
	( ) 5 - Strongly Agree

Telemedicine Confidence

9. **Before** participating in the learning activities, I was able to communicate effectively with a patient via telemedicine.	( ) 1 - Strongly Disagree
	( ) 2 - Disagree
	( ) 3 - Neutral
	( ) 4 - Agree
	( ) 5 - Strongly Agree
10. **After** participating in the learning activities, I am able to communicate effectively with a patient via telemedicine.	( ) 1 - Strongly Disagree
	( ) 2 - Disagree
	( ) 3 - Neutral
	( ) 4 - Agree
	( ) 5 - Strongly Agree
11. **Before** participating in the learning activities, I was able to take an effective patient history via telemedicine.	( ) 1 - Strongly Disagree
	( ) 2 - Disagree
	( ) 3 - Neutral
	( ) 4 - Agree
	( ) 5 - Strongly Agree
12. **After** participating in the learning activities, I am able to take an effective patient history via telemedicine.	( ) 1 - Strongly Disagree
	( ) 2 - Disagree
	( ) 3 - Neutral
	( ) 4 - Agree
	( ) 5 - Strongly Agree

13. **Before** participating in the learning activities, I was able to provide counseling to the patient effectively via telemedicine.	( ) 1 - Strongly Disagree
	( ) 2 - Disagree
	( ) 3 - Neutral
	( ) 4 - Agree
	( ) 5 - Strongly Agree
14. **After** participating in the learning activities, I am able to provide counseling to the patient effectively via telemedicine.	( ) 1 - Strongly Disagree
	( ) 2 - Disagree
	( ) 3 - Neutral
	( ) 4 - Agree
	( ) 5 - Strongly Agree
15. **Before** participating in the learning activities, I was able to communicate effectively with a multidisciplinary team via telemedicine.	( ) 1 - Strongly Disagree
	( ) 2 - Disagree
	( ) 3 - Neutral
	( ) 4 - Agree
	( ) 5 - Strongly Agree
16. **After** participating in the learning activities, I am able to communicate effectively with a multidisciplinary team via telemedicine.	( ) 1 - Strongly Disagree
	( ) 2 - Disagree
	( ) 3 - Neutral
	( ) 4 - Agree
	( ) 5 - Strongly Agree

Telemedicine Outlooks

17. **Before** participating in the learning activities, I thought telemedicine would help decrease geographic health care disparities.	( ) 1 - Strongly Disagree
	( ) 2 - Disagree
	( ) 3 - Neutral
	( ) 4 - Agree
	( ) 5 - Strongly Agree
18. **After** participating in the learning activities, I think telemedicine will help decrease geographic health care disparities.	( ) 1 - Strongly Disagree
	( ) 2 - Disagree
	( ) 3 - Neutral

	( ) 4 - Agree
	( ) 5 - Strongly Agree
19. **Before** participating in the learning activities, I thought telemedicine was an adequate alternative to in-person visits in health care.	( ) 1 - Strongly Disagree
	( ) 2 - Disagree
	( ) 3 - Neutral
	( ) 4 - Agree
	( ) 5 - Strongly Agree
20. **After** participating in the learning activities, I think telemedicine is an adequate alternative to in-person visits in health care.	( ) 1 - Strongly Disagree
	( ) 2 - Disagree
	( ) 3 - Neutral
	( ) 4 - Agree
	( ) 5 - Strongly Agree
21. **Before** participating in the learning activities, I thought telemedicine would help decrease health care disparities in rural areas.	( ) 1 - Strongly Disagree
	( ) 2 - Disagree
	( ) 3 - Neutral
	( ) 4 - Agree
	( ) 5 - Strongly Agree
22. **After** participating in the learning activities, I think telemedicine will help decrease health care disparities in rural areas.	( ) 1 - Strongly Disagree
	( ) 2 - Disagree
	( ) 3 - Neutral
	( ) 4 - Agree
	( ) 5 - Strongly Agree
23. **Before** participating in the learning activities, I thought telemedicine would help decrease health care disparities in urban areas.	( ) 1 - Strongly Disagree
	( ) 2 - Disagree
	( ) 3 - Neutral
	( ) 4 - Agree
	( ) 5 - Strongly Agree
24. **After** participating in the learning activities, I think telemedicine will help decrease health care disparities in urban areas.	( ) 1 - Strongly Disagree
	( ) 2 - Disagree

	( ) 3 - Neutral<br>( ) 4 - Agree<br>( ) 5 - Strongly Agree
25. **Before** participating in the learning activities, I thought telemedicine was most effective when performed in a multidisciplinary team setting.	( ) 1 - Strongly Disagree<br>( ) 2 - Disagree<br>( ) 3 - Neutral<br>( ) 4 - Agree<br>( ) 5 - Strongly Agree
26. **After** participating in the learning activities, I think telemedicine is most effective when performed in a multidisciplinary team setting.	( ) 1 - Strongly Disagree<br>( ) 2 - Disagree<br>( ) 3 - Neutral<br>( ) 4 - Agree<br>( ) 5 - Strongly Agree
27. **Before** participating in the learning activities, I was likely to incorporate telemedicine into my future practice	( ) 1 - Strongly Disagree<br>( ) 2 - Disagree<br>( ) 3 - Neutral<br>( ) 4 - Agree<br>( ) 5 - Strongly Agree
28. **After** participating in the learning activities, I am likely to incorporate telemedicine into my future practice	( ) 1 - Strongly Disagree<br>( ) 2 - Disagree<br>( ) 3 - Neutral<br>( ) 4 - Agree<br>( ) 5 - Strongly Agree
